# Rapid and Simultaneous Quantification of Levetiracetam and Its Carboxylic Metabolite in Human Plasma by Liquid Chromatography Tandem Mass Spectrometry

**DOI:** 10.1371/journal.pone.0111544

**Published:** 2014-11-06

**Authors:** Li-Ling Yeap, Yoke-Lin Lo

**Affiliations:** Department of Pharmacy, Faculty of Medicine, University of Malaya, Kuala Lumpur, Malaysia; SRI International, United States of America

## Abstract

A simple liquid chromatography tandem mass spectrometry method was developed and validated according to the guidelines of the US Food and Drug Administration and the European Medicines Agency for a simultaneous quantification of levetiracetam (LEV) and its metabolite, UCB L057 in the plasma of patients. A 0.050 mL plasma sample was prepared by a simple and direct protein precipitation with 0.450 mL acetonitrile (ACN) containing 1 µg/mL of internal standard (IS, diphenhydramine), then vortex mixed and centrifuged. A 0.100 mL of the clear supernatant was diluted with 0.400 mL water and well mixed. A 0.010 mL of the resultant solution was injected into an Agilent Zorbax SB-C18 (2.1 mm×100 mm, 3.5 µm) column with an isocratic elution at 0.5 mL/min using a mixture of 0.1% formic acid in water and ACN (40∶60 v/v). Detection was performed using an AB Sciex API 3000 triple quadrupole mass spectrometer, equipped with a Turbo Ion Spray source, operating in a positive mode: LEV at transition 171.1>154.1, UCB L057 at 172.5>126.1, and IS at 256.3>167.3; with an assay run time of 2 minutes. The lower limit of quantification (LLOQ) for both LEV and UCB L057 was validated at 0.5 µg/mL, while their lower limit of detection (LOD) was 0.25 µg/mL. The calibration curves were linear between 0.5 and 100 µg/mL for both analytes. The inaccuracy and imprecision of both intra-assay and inter-assay were less than 10%. Matrix effects were consistent between sources of plasma and the recoveries of all compounds were between 100% and 110%. Stability was established under various storage and processing conditions. The carryovers from both LEV and UCB L057 were less than 6% of the LLOQ and 0.13% of the IS. This assay method has been successfully applied to a population pharmacokinetic study of LEV in patients with epilepsy.

## Introduction

Levetiracetam [(S)-α-ethyl-2-oxo-1-pyrrolidine acetamide) or Keppra (UCB Inc, Smyrna, GA)] has been used as an adjunct or monotherapy in adults with partial onset seizures, with or without secondary generalization. Due to its efficacy and tolerability, the indications of levetiracetam have now been expanded to younger patients with a wider spectrum of epileptic syndromes such as myoclonic seizures and primary generalized tonic-clonic seizures [1].

Levetiracetam (LEV) is rapidly and completely absorbed after an oral administration. The drug has a linear pharmacokinetics with a minimum or no protein binding [2]. It does not undergo hepatic metabolism via cytochrome P450 and therefore has few drug-drug interactions [2]–[4]. Levetiracetam is converted to etiracetam carboxylic acid (UCB L057), an inactive metabolite via hydrolysis in the blood by beta-esterases [5]. About 66% of the absorbed dose is excreted unchanged in urine and 24% in its acid metabolite (UCB L057) form [4]. The elimination half-life of LEV is between 6 and 8 hours in adults with normal renal function, between 9 and 11 hours in elderly and 5 and 7 hours in children. The elimination half-life of LEV is prolonged in renal impairment, therefore dosage adjustment may be needed in patients with chronic kidney diseases or acute kidney injury.

Although LEV is recognized for its tolerability and ease of dosing due to its almost ideal pharmacokinetic profile, monitoring of serum or plasma concentrations of LEV may be useful in patients with altered physiological states; for example, in geriatric patients, pediatric patients or pregnant women; as well as in situations such as determining drug adherence, overdose or drug-induced adverse effects. Moreover, the co-administration with other inducers or inhibitors of cytochrome P450 enzymes has been reported to alter LEV serum concentrations [6]–[8].

A large between subject variability in the ratio of LEV serum concentrations to LEV dose/kg body weight; and LEV serum concentration-effect relationship has also been reported [6], [9]. Recently, Kauffman *et al.* [10] reported a probable association of LEV dose or plasma concentrations to mood disorders. The therapeutic range of LEV has not been distinctly defined, but a trough level of between 12 and 46 µg/mL or between 70 and 270 µmol/mL was suggested [11].

A number of laboratory methods such as immunoassay [12], high performance liquid chromatography with UV detection [9], [13]–[24], gas chromatography with mass spectrometry detection [25], [26], gas chromatography with nitrogen phosphorus detection [23], [27], [28], capillary electrophoresis with UV detection [29], high performance thin layer chromatography [15], high-performance liquid chromatography tandem mass spectrometry [15], [30]–[36] and ultra-performance liquid chromatography tandem mass spectrometry [37]–[40] have been described for measuring LEV in biological matrices. Some of these assay methods however, may require large sample volumes [14], [15], [17], [39], tedious extraction procedures using solid-phase extraction [13], [14], [16], [33] or liquid-liquid extraction [9], [14], [19], [22], [39] or a lengthy chromatographic run time of 10 minutes or longer, for an analysis of a single analyte [9], [16], [17], [19], [22]. Moreover, these assay methods mainly focus on the quantification of LEV, either alone or together with other antiepileptic drugs.

Although it is not crucial to measure an inactive metabolite in a pharmacokinetic-pharmacodynamic study, a falsely higher measured LEV concentrations may result if LEV was not separated either chromatographically or mass spectrometrically from UCB L057 during a quantification process [32]. Both compounds might co-elute as their molecular weights differ only by 1 mu and they also share a similar daughter ion of 126 mu which is often used for the quantification of LEV [15], [32]–[35], [37], [39], [40].

To date, there is only one published assay method that measures the plasma concentrations of LEV and UCB L057 simultaneously by altering the pH of the mobile phase using a gradient elusion [32]. Previous exploratory pharmacokinetic studies of LEV have employed two distinct analytical methods of GS-MS and LC-ESI-MS to quantify the plasma concentrations of LEV and UCB L057 respectively [27], [28], [41]. The objective of this present work is to develop and validate a simple LC-MS/MS method for a simultaneous quantification of LEV and UCB L057 in the plasma of patients treated with LEV for seizure control in a population pharmacokinetic study.

## Materials and Methods

### 2.1 Reagents, internal standard, calibrators and quality control samples

**Figure 1:**
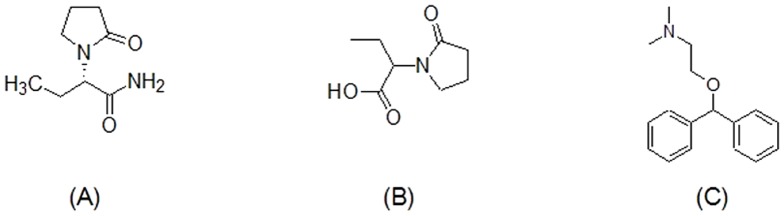
Molecular structures of (A) Levetiracetam (molecular weight 170.2), (B) Etiracetam carboxylic acid (molecular weight 171.2) and (C) Diphenhydramine (molecular weight 255.4).

Pure compounds of LEV (purity ≥98%) and UCB L057 (purity 98%) were purchased from Sigma-Aldrich (Missouri, USA) and Toronto Research Chemical (Ontario, Canada), respectively. Internal standard (IS), diphenhydramine (DPH) is a gift from Pharmaniaga Ltd (Selangor, Malaysia). The molecular structures of LEV, UCB L057 and DPH are displayed in Figure 1.

HPLC-grade acetonitrile (ACN) and HPLC-grade methanol were purchased from Fisher Scientific (Leicester, UK). Formic acid 98% was purchased from Acros Organic (New Jersey, USA). All water was distilled and purified with a Sartorius Reverse Osmosis Arium, RO 61316 system and Elga Purelab UHQ, UHQ-PS-MK3 (18 MΩ). Drug-free, human plasma was supplied by the Transfusion Unit of University of Malaya Medical Centre (Kuala Lumpur, Malaysia).

Both the stock solutions of LEV and UCB L057 were prepared at 5.000 mg/mL and the working solution of IS at DPH 1.000 mg/mL in methanol/UHQ water (50∶50 v/v). Separate stock solutions were used to prepare calibration standards (references) and quality control (QC) samples. A set of calibrators at concentrations of 0.5, 5, 10, 40, 60, 80 and 100 µg/mL and QC standards at concentrations of 1.5, 50 and 90 µg/mL, were prepared with each series, by spiking pooled blank human plasma with an appropriate stock solution. The amount of stock solutions added to the plasma did not exceed 3% of the total volume.

### 2.2 LC-MS/MS instrumentation and conditions

Analysis of LEV and UCB L057 was performed using an Agilent 1100 HPLC system equipped with a binary solvent pump, an autosampler, a column oven and a degasser (Agilent Technologies, Palo Alto, CA, USA). The HPLC system was connected to an AB Sciex API 3000 triple quadrupole mass spectrometer (AB Sciex Instruments, New Jersey, USA) equipped with a Turbo Ion Spray source. Chromatographic data analysis was performed by Analyst software (Version 1.4.2).

The chromatographic separation of LEV, UCB L057 and IS was performed on an Agilent Zorbax SB-C18 (2.1 mm×100 mm, 3.5 µm) column mounted with a Supelco replacement frit (0.5 µm pore size) with an isocratic elution. The mobile phase consists of a mixture of 0.1% formic acid in water and ACN (40∶60 v/v). The flow rate was kept constant at 0.5 mL/min while the temperature of the column oven was maintained at 35°C. The injection volume is 0.010 mL and the assay run time is 2 minutes.

Mass spectral analysis was performed on a positive electrospray ionization mode with the following parameters: source temperature of 450°C, ion spray voltage at 5,000 V, nitrogen as the nebulizer gas. The flow of nebulizing gas, curtain gas, and collision gas were at instrument settings of 8, 8, and 4, respectively. The optimized settings of mass spectrometer voltage and the retention time for each analyte are presented in Table 1.

**Table 1:** Optimized mass spectrometer voltage settings including MS/MS transitions for all analytes evaluated.

Analyte	Q1/Q3 transitions (m/z), precursor ion> product ion	DP (V)	FP (V)	CE (V)	CXP (V)	EP (V)	Dwell time (msec)	Retention time (min)
LEV	171.1>154.1	18	213	11	15	10	100	0.58
UCB L057	172.5>126.1	26	213	18	15	10	100	0.61
IS	256.3>167.3	17	213	20	15	10	100	0.82

DP  =  declustering potential, FP  =  focusing potential, CE  =  collision energy, CXP  =  collision cell exit potential and EP  =  entrance potential.

### 2.3 Sample preparation

Samples were prepared by a simple and direct protein precipitation. To 0.050 mL of sample was added 0.450 mL of precipitating solution containing 1 µg/mL of IS in ACN. The mixture was vortex mixed for 20 s, and then centrifuged at 15,900×*g* for 5 min. A 0.100 mL of the clear supernatant was transferred into a clean microcentrifuge tube containing 0.400 mL of UHQ water. A 0.010 mL of the final mixture in a dilution of 1∶50, was then directly introduced into the chromatographic system after mixing.

### 2.4 Method validation

The assay method was validated according to the requirements as outlined in guidelines established by the US Food and Drug Administration (US FDA) [42] and the European Medicines Agency (EMA) [43].

### 2.5 Collection and storage of plasma samples from patients

This assay method was applied to an analysis of LEV and its metabolite in plasma samples collected from adult patients with epilepsy. These patients were recruited to participate in a population pharmacokinetic study at the University of Malaya Medical Centre (UMMC) in Malaysia. The study protocol was reviewed and approved by the UMMC Medical Ethics Committee (Approval Reference No. 890.31) and was carried out in accordance with the Declaration of Helsinki and guidelines for Good Clinical Practice. Written informed consent was obtained from a patient or a next-of-kin of a patient before enrollment in the study. Blood samples were collected from an intravenous cannula before an oral prescribed LEV dose and at 15, 30, 60 min, 2, 3.5 and 5 h after an oral administration. Venous blood samples in lithium heparin tubes were centrifuged at 900×*g* for 10 min and the plasma were extracted and transferred into clean cryo vials and immediately stored at −20°C until analysis.

## Results and Discussion

### 3.1 Method development

As LEV lacks chromophores, the utilization of MS/MS detection method may be more feasible [19]. The quantification of LEV using mass spectrometry however, may be affected by the fact that both LEV and its carboxylic metabolite share the same product ion of the highest abundance (Q1/Q3 transitions of 171.1>126.1 for LEV and 172.5>126.1 for UCB L057). Additionally, both LEV and UCB L057 may also co-elute and thus giving rise to falsely higher measured LEV concentrations [32]. Prior to this analytical method report, there was only one other assay method that enables the separation and simultaneous quantification of LEV and UCB L057 [32]. The authors separated LEV from its carboxylic metabolite chromatographically by having a mobile phase of pH close to 2.5 with 0.1% formic acid. This acid condition keeps the metabolite in a non ionized form to retain it longer in an analytical column.

**Figure 2:**
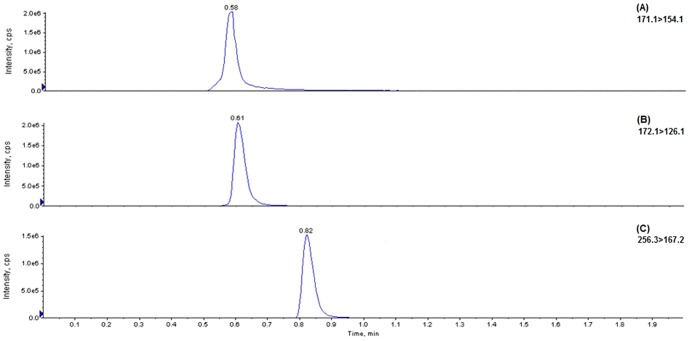
Chromatograms of extracted plasma spiked with (A) LEV at 50 µg/mL [QC medium level], (B) UCB L057 at 50 µg/mL [QC medium level] and (C) IS.

The same product ions for both LEV and UCB L057 were not selected. The product ion of the second highest abundance for LEV (Q1/Q3 transitions of 171.1>154.1) was chosen for multiple reaction monitoring (MRM) instead. There was no compromise to the sensitivity for LEV. All analytes were eluted in less than 1 min, permitting an injection-to-injection cycle time of 2.0 min. The developed method provides a stable retention time for all analytes without the need to stringently control the pH of the mobile phase. Representative chromatograms of medium quality control (QCM) at 50 µg/mL of both LEV and UCB L057 as well as IS, with their respective retention time, are shown in Figure 2.

### 3.2 Method validation

#### 3.2.1 Limit of detection (LOD) and lower limit of quantification (LLOQ)

The LOD and LLOQ were expressed as a ratio of signal to noise (S/N) ≥3 and ≥5, respectively. The LLOQs for both LEV and UCB L057 were validated at 0.5 µg/mL, while the LOD was 0.25 µg/mL for both analytes.

#### 3.2.2 Selectivity

Six different sources of blank plasma were processed to check for interference from endogenous compounds. The effects of hemolyzed blood and commonly used additives in blood tubes such as lithium heparin, ethylenediaminetetraacetic acid (EDTA) and citric acid were investigated. When the chromatograms of extracted drug-free plasma were overlaid with those of extracted spiked plasma samples, no significant peak interferences were observed at the respective retention times of LEV and UCB L057. The calculated percentage interferences were less than 15% of the LLOQ for both analytes and 0.2% for the IS, which is acceptable according to the requirements stated in EMA [43]. Similarly, no peak interferences were observed for the studied hemolyzed matrix and additives matrix containing lithium heparin, EDTA and citric acid.

#### 3.2.3 Calibration curve and linearity

A 1/x^2^ weighted linear regression model was performed using the Analyst software to construct the calibration curve of both LEV and UCB L057. The calibration curves were linear over a working range between 0.5 and 100 µg/mL for both analytes and the regression coefficients (r^2^) of all calibration curves were more than 0.990. The current calibrated range is clearly wider than the recommended LEV therapeutic range of 12 to 46 µg/mL [11]. Previous pharmacokinetic studies involving an administration of a single, low dose of LEV reported low UCB L057 concentrations of less than 5 µg/mL [27], [28], [41]. In the present assay method, a wider calibration range for UCB L057 was also determined to support future pharmacokinetic studies on LEV in various clinical settings; for example, an unexpected accumulation of UCB L057 due to prolonged elimination in patients with renal impairment or when larger doses of LEV are used in status epilepticus.

#### 3.2.4 Accuracy and precision

The accuracy and precision of the assay method were established at four concentration points of the calibration curve: at 0.5 µg/mL (QC at LLOQ, QCLLOQ), 1.5 µg/mL (QC at low level, QCL), 50 µg/mL (QC at medium level, QCM) and 90 µg/mL (QC at high level, QCH). The intra-assay accuracy and precision were determined by measuring seven replicates of each of the spiked QC samples in a single analytical run. For inter-assay accuracy and precision, the calculated concentrations for all 4 levels of QC samples from 7 independent cycles were used. The accuracy and precision of both intra-assay and inter-assay were all within the acceptance criteria set by the US FDA and the EMA (Table 2).

**Table 2:** The accuracy and precision of intra-assay and inter-assay for each analyte.

Compounds	Level	Intra-assay (7 samples)			Inter-assay (7 runs)		
		Accuracy (%)	Imprecision		Accuracy (%)	Imprecision	
			Measured (µg/mL)	CV (%)		Measured(µg/mL)	CV (%)
LEV	QCLLOQ (0.5 µg/mL)	94.51	0.47±0.04	9.13	100.21	0.50±0.04	8.14
	QCL (1.5 µg/mL)	102.80	1.54±0.08	5.15	104.41	1.57±0.12	7.37
	QCM (50 µg/mL)	99.80	49.81±3.81	7.65	98.94	49.51±3.77	7.62
	QCH (90 µg/mL)	108.86	98.11±3.65	3.72	97.80	88.03±8.21	9.33
UCB L057	QCLLOQ (0.5 µg/mL)	92.13	0.46±0.02	4.87	98.52	0.49±0.04	8.71
	QCL (1.5 µg/mL)	100.51	1.51±0.09	5.85	106.15	1.59±0.13	8.31
	QCM (50 µg/mL)	104.36	52.20±2.46	4.71	100.82	50.43±3.77	7.47
	QCH (90 µg/mL)	99.11	89.29±6.39	7.16	94.50	85.03±5.86	6.89

*QCLOQ is QC at LLOQ concentration, QCL is QC at low concentration, QCM is QC at medium concentration and QCH is QC at high concentration. LEV is levetiracetam and UCB L057 is its carboxylic metabolite. CV is coefficient of variation and is expressed in percentage (%).*

#### 3.2.5 Recovery and matrix effects

Recovery represents the extraction efficiency of an analytical method while matrix effects are the combined interference with the ionization process in a mass spectrometer of all components of a sample other than the analytes of interest. Extracted pooled blank plasma (6 replicates) was used for the assessment of recovery at QCL, QCM and QCH while matrix effects were investigated with 7 lots of individual blank plasma at QCL and QCH, and calculated for each lot of matrix for each analyte. Both LEV and UCB L057 were spiked at 1∶50 ratio into extracted blank plasma and blank reagent (ACN:H_2_O, 18∶82, v/v) containing IS and were used as a comparison for the assessment of recovery and matrix effects.

The following formulas were applied to calculate the recovery (RE) and the matrix effect (ME):
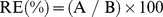


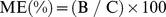
where A is the peak height of a pre extraction spiked standard, B is the peak height of a post extraction spiked standard at a 1∶50 dilution ratio, and C is the peak height of analyte spiked in a standard solution at a 1∶50 dilution ratio.

As shown in Table 3, the accuracy and imprecision for all analytes in recovery study were within the acceptance limits of the US FDA [42]. The matrix effects remained consistent between various sources of plasma with a CV of less than 15% and therefore would not adversely affect the accuracy and precision of the assay method.

**Table 3:** Recovery and matrix effects for each analyte.

Level	Nominal conc. (µg/mL)	Compounds	Recovery (n = 6)		Matrix effects (n = 7)	
			Percentage	CV (%)	Percentage	CV (%)
QCL	1.5	LEV	107.72	4.60	100.97	13.38
		UCB L057	103.08	5.81	94.02	13.36
		IS	100.93	2.30	93.68	8.30
QCM	50	LEV	105.30	3.12	-	-
		UCB L057	105.07	3.81	-	-
		IS	101.74	2.00	-	-
QCH	100	LEV	101.12	2.14	95.99	14.90
		UCB L057	102.44	1.60	92.60	13.44
		IS	100.12	4.37	110.06	12.76

*QCLOQ is QC at LLOQ concentration, QCL is QC at low concentration, QCM is QC at medium concentration and QCH is QC at high concentration. LEV is levetiracetam and UCB L057 is its carboxylic metabolite. CV is coefficient of variation and is expressed in percentage (%).*

#### 3.2.6 Stability

The stability of LEV and UCB L057 were carried out by comparing the measured concentrations of samples under short-term or long-term storage, or various conditions resembling the actual sample preparation before analysis, against those of freshly prepared samples. No significant difference in concentrations was observed between these batches (Table 4).

**Table 4:** Stability study for each analyte.

Stability test	Storage condition	Matrix	LEV			UCB L057		
			QCL [1.5 µg/mL]	QCM [50 µg/mL]	QCH [90 µg/mL]	QCL [1.5 µg/mL]	QCM [50 µg/mL]	QCH [90 µg/mL]
Stock solution (n = 6)	6 hours at RT	Reagent	-	+5.39	-	-	+1.71	-
	2 months in -20°C	Reagent	-	−4.42	-	-	−9.58	-
Bench top (n = 4)	4 hours at RT	Plasma	+2.92	-	-8.89	−1.50	-	−11.08
Freeze-thaw (n = 4)	Freeze-thaw (3 cycles)	Plasma	+0.17	-	−3.44	+6.00	-	+1.42
Long term (n = 4)	1 week in −20°C	Plasma	+7.11	-	+2.74	−8.89	-	+0.63
	2 months in −20°C	Plasma	+8.67	-	−2.19	−3.33	-	−1.64
Auto-sample (n = 4)	24 hours at RT	Extracted plasma	+1.56	-	+9.86	−9.11	-	−2.97

*QCLOQ is QC at LLOQ concentration, QCL is QC at low concentration, QCM is QC at medium concentration and QCH is QC at high concentration. LEV is levetiracetam and UCB L057 is its carboxylic metabolite. CV is coefficient of variation and is expressed in percentage (%).RT is room temperature. The presented data in this table are calculated as % deviation (% CV).*

*Analytes and IS are spiked into reagent at 1∶50 dilution ratio.

#### 3.2.7 Carryover

The carryover effect was evaluated by injecting the highest concentration of the calibration standard (100 µg/mL) followed by a blank reagent (ACN:H_2_O, 18∶82, v/v) for 3 replicates. The carryover from both LEV and UCB L057 was acceptable at less than 6% of the LLOQ and 0.13% of the IS.

#### 3.2.8 Dilution integrity

Since the plasma concentrations of LEV and UCB L057 from patient samples may exceed the highest established linearity range, sample dilution procedure may be necessary. Dilution integrity of 1∶2 and 1∶4 was investigated by diluting a 0.025 mL of spiked plasma sample at two folds the concentration of QCH (180 µg/mL) of LEV and UCB L057 with 0.025 mL of blank plasma or a 0.0125 mL of this spiked plasma with 0.0375 mL of blank plasma, respectively before extraction. The inaccuracy and imprecision were within the limit of 15%.

### 3.3 Clinical applications

This validated assay method was applied to a population pharmacokinetic study to determine the concentrations of LEV and UCB L057 simultaneously in 318 plasma samples contributed by 50 patients aged between 18 and 64 years, weighing between 38.6 and 93 kg and receiving LEV doses between 0.5 and 4.5 g per day. The simultaneous measurement of plasma concentrations of the parent compound of LEV and its metabolite allows a more in depth evaluation of the pharmacokinetic profile of LEV. The extent of conversion from the parent compound to its metabolite as well as the systemic eliminations of both compounds in patients with various pathophysiological conditions can then be assessed.

**Figure 3:**
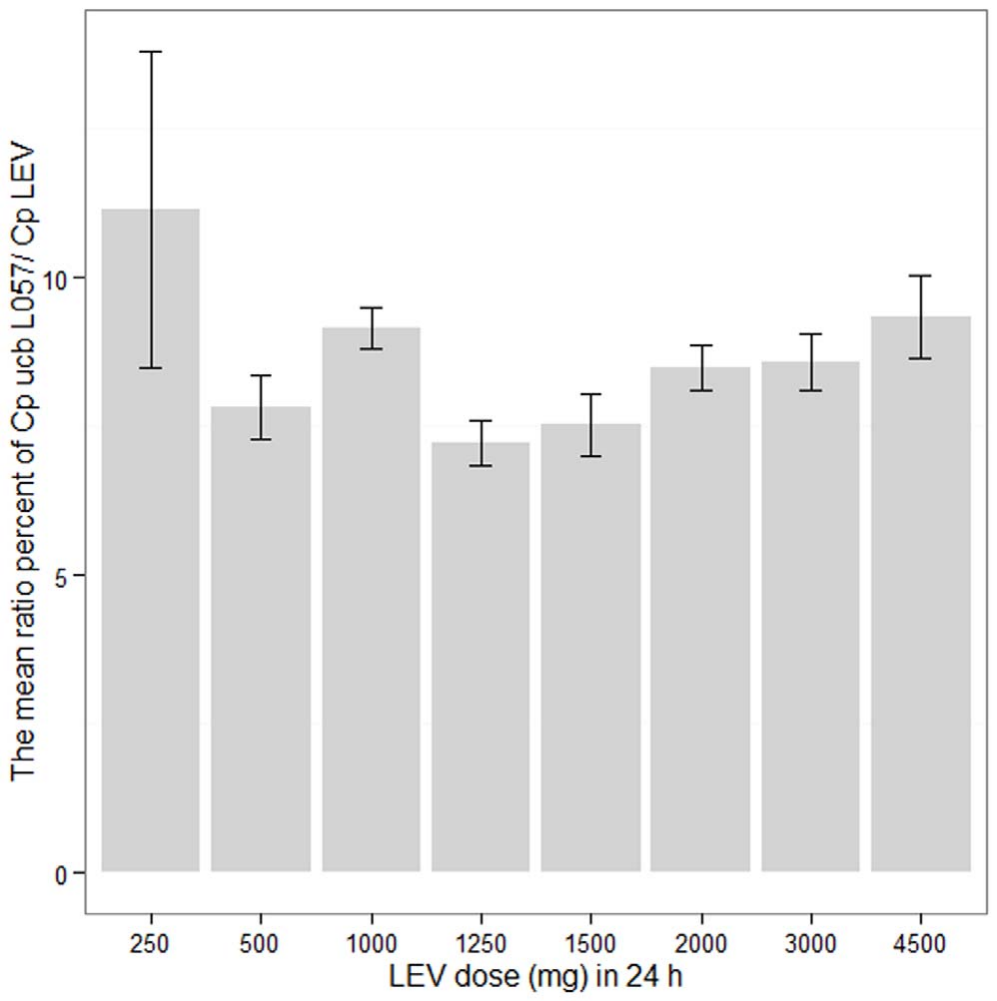
Mean and standard error of mean (SEM) of the ratio percent of plasma concentrations of UCB L057/LEV at various dosing regimens.

**Figure 4:**
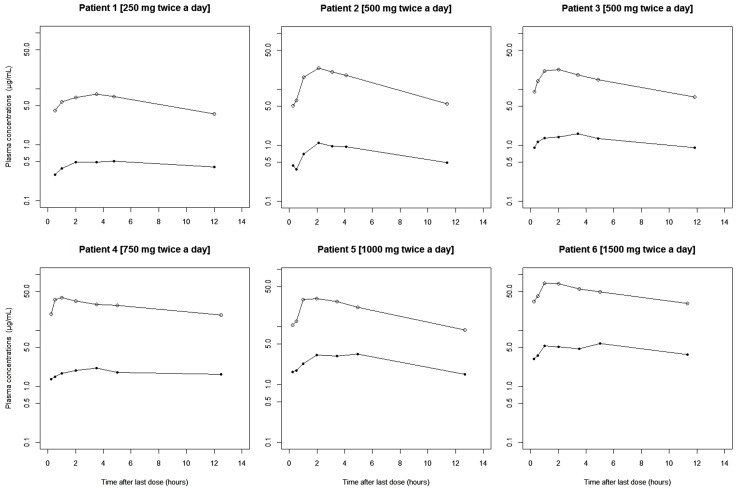
Representative semi-log plots of plasma concentrations of LEV (○) and UCB L057 (•) versus time after last dose of 6 patients with epilepsy on maintenance dose of oral LEV.

The mean plasma concentrations of UCB L057 were low compared with that of LEV. Figure 3 depicts the mean and the standard error of mean (SEM) of the ratio percent of plasma concentrations of UCB L057/LEV at various dosing regimens. The mean ratio percent of plasma concentrations of UCB L057 at T_max_ of LEV over C_max_ of LEV was 6.7% (SD ±2.2%). This value is higher than the 3% [41], [44] or 5% [45] value reported in previous LEV pharmacokinetic studies in children where a single dose or multiple doses of LEV were administered. The difference in our study may be due to a different assay method used, a more mature liver metabolic function in adults and the co-administration of other antiepileptic agents that are enzyme inducers. The profiles of plasma concentrations versus time after last dose from 6 sample patients receiving various dosing regimens of LEV are displayed in Figure 4.

## Conclusion

A simple method for the simultaneous determination of plasma concentrations of LEV and UCB L057 using LC-MS/MS was developed and validated to be used in a population pharmacokinetic study or other clinical studies in patients with epilepsy. The current assay method requires a relatively small sample volume of 0.050 mL, involves a simple and fast sample preparation, as well as a short assay run time of 2 min. This assay method has also been subjected to thorough validation processes and meets the standards as outlined by the requirements of both the US FDA and the EMA.

## Supporting Information

Table S1 **Mean, SD and SEM of plasma concentrations of LEV and UCB L057, with corresponding total daily LEV dose.**
(DOCX)

Table S2 **Data of the pre- and post-dose plasma concentrations of LEV and UCB L057 from 6 patients with epilepsy as presented in** **Figure 4** **.**
(DOCX)
