# Supplementary material for: Rapid and Simultaneous Quantification of Levetiracetam and Its Carboxylic Metabolite in Human Plasma by Liquid Chromatography Tandem Mass Spectrometry
Source: PLoS One. 2014 Nov 6;9(11):e111544. doi: 10.1371/journal.pone.0111544 (PMC4223074; doi:10.1371/journal.pone.0111544)
Supplement: Table S1 — Mean, SD and SEM of plasma concentrations of LEV and UCB L057, with corresponding total daily LEV dose. (DOCX) [file pone.0111544.s001.docx]

**Table S1. Mean, SD and SEM of plasma concentrations of LEV and UCB L057, with corresponding total daily LEV dose**

| LEV dose (mg/day) | Plasma concentration of LEV (µg/mL) | | | | Plasma concentration of UCB L057 (µg/mL) | | | |
| --- | --- | --- | --- | --- | --- | --- | --- | --- |
|  | Mean | SD | N | SEM | Mean | SD | N | SEM |
| 250 | 1.780 | 0.526 | 5 | 0.235 | 0.190 | 0.093 | 5 | 0.041 |
| 500 | 8.049 | 3.375 | 49 | 0.482 | 0.580 | 0.248 | 49 | 0.035 |
| 1000 | 16.460 | 7.024 | 107 | 0.679 | 1.362 | 0.431 | 107 | 0.042 |
| 1250 | 31.859 | 18.474 | 14 | 4.937 | 2.164 | 1.211 | 14 | 0.324 |
| 1500 | 26.530 | 14.361 | 21 | 3.134 | 1.729 | 0.588 | 21 | 0.128 |
| 2000 | 25.800 | 13.070 | 72 | 1.540 | 2.004 | 0.912 | 72 | 0.107 |
| 3000 | 36.329 | 17.154 | 21 | 3.743 | 2.982 | 1.356 | 21 | 0.296 |
| 4500 | 52.200 | 8.280 | 7 | 3.129 | 4.760 | 0.408 | 7 | 0.154 |

*LEV = levetiracetam; UCB L057 = etiracetam carboxylic acid; N = No. of the concentration points; SEM = standard error of the mean*
