# Supplementary material for: Rapid and Simultaneous Quantification of Levetiracetam and Its Carboxylic Metabolite in Human Plasma by Liquid Chromatography Tandem Mass Spectrometry
Source: PLoS One. 2014 Nov 6;9(11):e111544. doi: 10.1371/journal.pone.0111544 (PMC4223074; doi:10.1371/journal.pone.0111544)
Supplement: Table S2 — Data of the pre- and post-dose plasma concentrations of LEV and UCB L057 from 6 patients with epilepsy as presented in Figure 4 . (DOCX) [file pone.0111544.s002.docx]

**Table S2. Data of the pre- and post-dose plasma concentrations of LEV and UCB L057 from 6 patients with epilepsy as presented in Figure 4**

| **Patient ID** | **Dose of LEV per day (mg)** | **Time after dose (h)** | **LEV concentrations (mcg/mL)** | | **UCB L057 concentrations (mcg/mL)** | |
| --- | --- | --- | --- | --- | --- | --- |
| 1 | 500 | 0 | . | . | |  |
| 1 |  | 0.5 | 4.08 | 0.29 | |  |
| 1 |  | 1.0 | 5.89 | 0.38 | |  |
| 1 |  | 2.0 | 7.04 | 0.49 | |  |
| 1 |  | 3.5 | 8.01 | 0.49 | |  |
| 1 |  | 4.8 | 7.26 | 0.50 | |  |
| 1 |  | 12.0 | 3.57 | 0.40 | |  |
| 2 | 1000 | 0 | . | . | |  |
| 2 |  | 0.3 | 5.09 | 0.44 | |  |
| 2 |  | 0.5 | 6.33 | 0.37 | |  |
| 2 |  | 1.1 | 16.30 | 0.69 | |  |
| 2 |  | 2.1 | 24.00 | 1.10 | |  |
| 2 |  | 3.1 | 20.50 | 0.96 | |  |
| 2 |  | 4.1 | 17.70 | 0.93 | |  |
| 2 |  | 11.4 | 5.54 | 0.48 | |  |
| 3 | 1000 | 0 | . | . | |  |
| 3 |  | 0.3 | 9.12 | 0.90 | |  |
| 3 |  | 0.5 | 14.00 | 1.15 | |  |
| 3 |  | 1.0 | 21.20 | 1.34 | |  |
| 3 |  | 2.0 | 22.50 | 1.40 | |  |
| 3 |  | 3.4 | 18.20 | 1.59 | |  |
| 3 |  | 4.9 | 15.00 | 1.32 | |  |
| 3 |  | 11.9 | 7.22 | 0.91 | |  |
| 4 | 1500 | 0 | . | . | |  |
| 4 |  | 0.3 | 19.60 | 1.34 | |  |
| 4 |  | 0.5 | 35.60 | 1.48 | |  |
| 4 |  | 1.0 | 38.40 | 1.72 | |  |
| 4 |  | 2.0 | 33.70 | 1.92 | |  |
| 4 |  | 3.5 | 29.00 | 2.12 | |  |
| 4 |  | 5.0 | 28.30 | 1.79 | |  |
| 4 |  | 12.5 | 18.70 | 1.64 | |  |
| 5 | 2000 | 0 | . | . | |  |
| 5 |  | 0.3 | 10.60 | 1.66 | |  |
| 5 |  | 0.5 | 12.50 | 1.76 | |  |
| 5 |  | 1.0 | 29.40 | 2.30 | |  |
| 5 |  | 2.0 | 30.90 | 3.26 | |  |
| 5 |  | 3.4 | 27.10 | 3.08 | |  |
| 5 |  | 4.9 | 21.70 | 3.33 | |  |
| 5 |  | 12.7 | 8.79 | 1.50 | |  |
| 6 | 3000 | 0 | . | . | |  |
| 6 |  | 0.25 | 32.80 | 3.12 | |  |
| 6 |  | 0.5 | 40.90 | 3.54 | |  |
| 6 |  | 1.0 | 70.20 | 5.26 | |  |
| 6 |  | 2.0 | 68.60 | 5.11 | |  |
| 6 |  | 3.5 | 55.10 | 4.71 | |  |
| 6 |  | 5.0 | 48.60 | 5.89 | |  |
| 6 |  | 11.3 | 30.70 | 3.68 | |  |
